# Restoring the Airway Barrier: How Targeting Eosinophilic Inflammation Shapes Epithelial Health and Clinical Remission in Asthma

**DOI:** 10.3390/diagnostics16182920

**Published:** 2026-09-10

**Authors:** Sabina Gasperovic, Andrius Januskevicius, Kestutis Malakauskas

**Affiliations:** 1 Department of Pulmonology, Lithuanian University of Health Sciences, LT-44307 Kaunas, Lithuania; kestutis.malakauskas@lsmu.lt; 2 Laboratory of Pulmonology, Department of Pulmonology, Lithuanian University of Health Sciences, LT-44307 Kaunas, Lithuania; andrius.januskevicius@lsmu.lt

**Keywords:** asthma, eosinophilic inflammation, airway epithelium, epithelial barrier dysfunction, biomarkers, asthma remission

## Abstract

Asthma is a heterogeneous chronic airway disease in which eosinophilic inflammation represents a major type 2 endotype. The airway epithelium plays a central role in disease pathobiology by regulating barrier integrity, immune signaling, and tissue repair. In eosinophilic asthma, interactions between eosinophils and epithelial cells are associated with epithelial barrier disruption, altered differentiation, mucus hypersecretion, and airway remodeling. This review summarizes current evidence on the role of eosinophils in epithelial dysfunction and discusses biomarkers of epithelial injury, including epithelial-derived alarmins, chemokines, secretory proteins, and structural junctional markers. These biomarkers reflect different aspects of epithelial activation, injury, and repair and may contribute to improved characterization of disease mechanisms and inflammatory endotypes. The effects of biologic therapies targeting eosinophilic inflammation are also reviewed. While these treatments are associated with improved clinical outcomes and reduced eosinophil levels, available evidence indicates that epithelial alterations and remodeling processes may persist despite treatment. Overall, integrating epithelial biomarkers with clinical and inflammatory parameters may provide additional insight into disease activity and support more comprehensive assessment of asthma beyond conventional measures.

## 1. Introduction

Asthma is one of the most common chronic respiratory diseases and affects more than 300 million people worldwide [1]. It is characterized by chronic airway inflammation, airway remodeling, and airway hyperresponsiveness (AHR), with symptoms such as wheezing, shortness of breath, chest tightness, and cough that vary over time and are associated with variable airflow obstruction [2]. Asthma is a heterogeneous disease and is commonly divided into type 2-high and type 2-low endotypes. Eosinophilic asthma is characterized by increased eosinophil levels in the airways, peripheral blood, and sputum. Despite major advances in treatment, the persistent global burden of asthma indicates that important aspects of its pathogenesis remain incompletely understood. Development of asthma is influenced by interactions between genetic susceptibility and environmental exposures, including allergens and pathogens, which contribute to chronic airway inflammation [3]. This process involves interactions between immune cells and structural cells of the airway mucosa [3]. Eosinophilic inflammation, particularly in allergic and severe asthma, is considered an important contributor to epithelial changes and airway dysfunction [4]. The airway epithelium forms the first barrier between the respiratory tract and the external environment. It consists of different cell types, including ciliated epithelial cells, goblet cells, basal cells, and neuroendocrine cells, which contribute to mucociliary clearance, barrier integrity, and immune regulation [5]. Increasing evidence suggests that epithelial dysfunction is involved in asthma pathophysiology and includes both structural abnormalities and altered immunoregulatory function [6]. Direct assessment of epithelial integrity remains limited in clinical practice. Because of this, biomarkers of epithelial injury have gained increasing attention. These biomarkers may reflect epithelial damage, activation, or repair processes [7]. Several epithelial-derived mediators detectable in blood or airway samples, including nasal epithelial samples, have been proposed as indicators of airway barrier dysfunction and epithelial activation [8]. Changes in their levels may reflect ongoing epithelial stress, impaired barrier integrity, or processes related to epithelial repair and regeneration. They may also provide information about disease activity and inflammatory endotypes that is not always captured by conventional clinical measures.

Current asthma treatment includes inhaled steroids, long-acting muscarinic antagonists, long-acting β-agonists, leukotriene receptor antagonists, and biologic therapies. Eosinophilic asthma represents one of the more severe forms of disease and often remains difficult to control. Biologic therapies developed for severe eosinophilic asthma include monoclonal antibodies targeting the IL-5 pathway, including mepolizumab, reslizumab, and benralizumab [4,9].

The introduction of biologic therapies targeting eosinophilic inflammation has significantly improved outcomes in patients with severe asthma. The concept of asthma remission has changed over time and now includes not only symptom control but also longer-term suppression of inflammatory activity and disease-related changes. Restoration of epithelial integrity has therefore been proposed as a potential biological correlate of long-term clinical remission [10,11]. Clinical remission in asthma is generally defined by sustained symptom control, absence of exacerbations, stable lung function, and no use of oral corticosteroids for asthma over an extended period [10,12]. However, clinical improvement does not necessarily indicate complete resolution of the underlying disease processes. Inflammatory activity and structural airway abnormalities can remain despite good clinical control. Increasing attention has shifted toward biological remission, which refers to suppression of disease-related inflammatory pathways and reduction of ongoing airway tissue changes [13]. Restoration of epithelial barrier function and reduction of epithelial injury markers may represent evidence of biological remission beyond conventional clinical endpoints.

This review aims to summarize current evidence on the role of eosinophils in airway epithelial dysfunction, to discuss emerging biomarkers of epithelial injury in asthma, and to explore whether epithelial restoration is associated with clinical remission, particularly in the context of biologic therapies.

## 2. The Role of Eosinophils in Epithelial Dysfunction

### 2.1. Eosinophil-Driven Regulation of Epithelial Differentiation and Barrier Dysfunction

**Figure 1:**
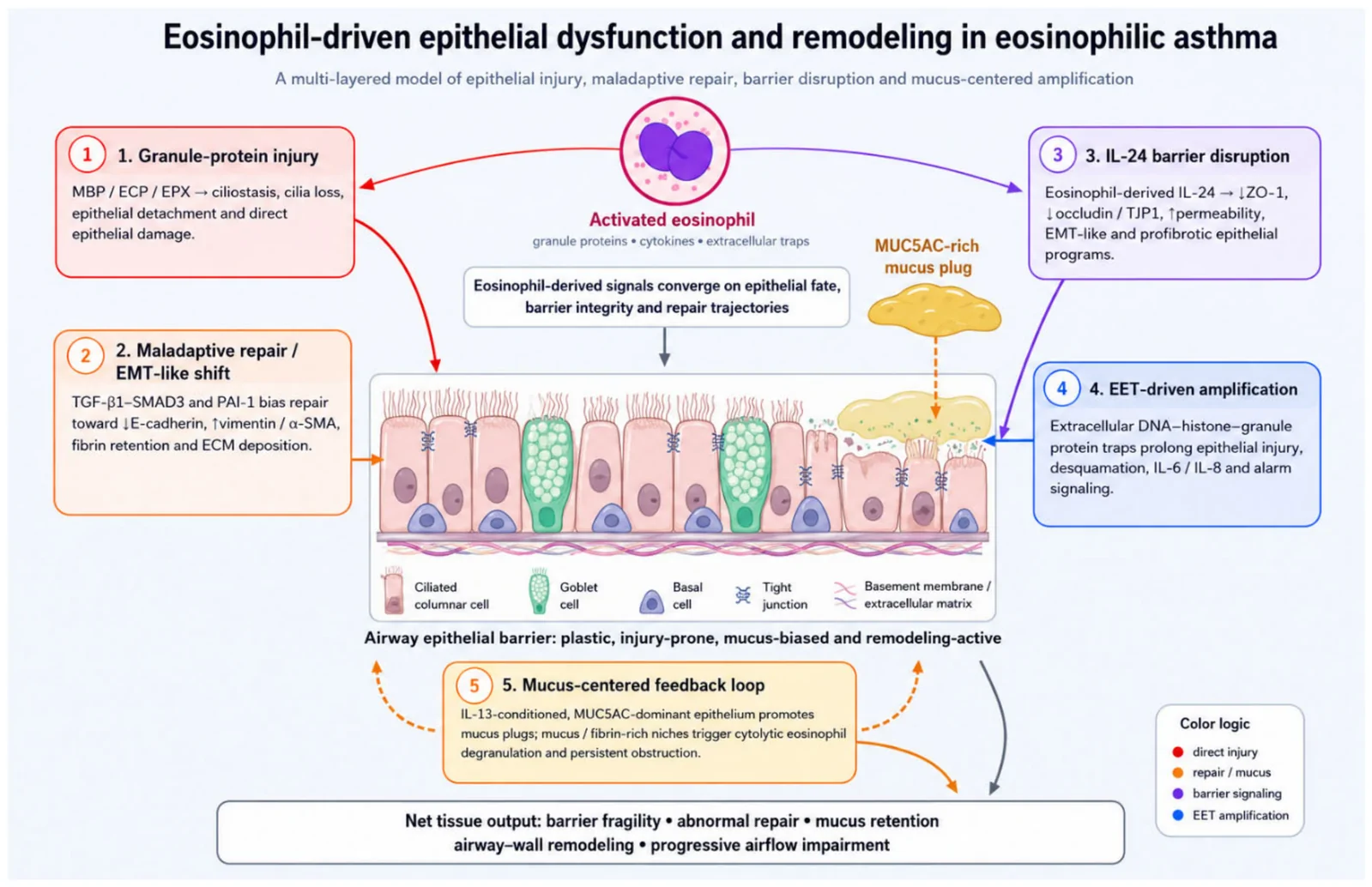
Eosinophil-mediated epithelial dysfunction and remodeling in eosinophilic asthma. Activated eosinophils contribute to airway epithelial dysfunction through several interrelated mechanisms: their granule proteins, including MBP, ECP, and EPX, directly damage the epithelium, inhibit ciliary function, and promote epithelial cell detachment and loss of barrier integrity [14,15]. In addition, eosinophils alter the direction of epithelial repair by promoting TGF-β1/SMAD3 and PAI-1-related processes, downregulating E-cadherin expression, increasing vimentin/α-SMA expression, fibrin accumulation, and ECM deposition, thereby skewing epithelial repair toward an EMT-like, pro-remodeling phenotype [15,16]. Eosinophil-derived IL-24 further weakens the epithelial barrier by downregulating ZO-1, occludin, and TJP1 expression, increasing epithelial permeability, and activating profibrotic programs, while EETs maintain prolonged epithelial damage, desquamation, IL-6/IL-8 release, and amplification of type 2 inflammation [21,22]. At the same time, eosinophil-epithelial interactions promote a MUC5AC-dominant secretory phenotype, goblet-cell metaplasia, mucus hyperproduction, and the formation of MUC5AC-rich mucus plugs; such mucus- and fibrin-rich niches can further activate eosinophils, sustaining cytolytic degranulation, mucus retention, airway wall remodeling, and progressive airflow obstruction [14,15,28]. α-SMA, alpha-smooth muscle actin; ECP, eosinophil cationic protein; ECM, extracellular matrix; EETs, eosinophil extracellular traps; EMT, epithelial–mesenchymal transition; EPX, eosinophil peroxidase; IL, interleukin; MBP, major basic protein; MUC5AC, mucin 5AC; PAI-1, plasminogen activator inhibitor-1; SMAD3, mothers against decapentaplegic homolog 3; TGF-β1, transforming growth factor-β1; TJP1, tight junction protein 1; ZO-1, zonula occludens-1.

Eosinophils should be regarded not only as terminal effector cells of type 2 inflammation but also as local regulators of airway epithelial fate in asthma [14,15]. Their importance lies in the fact that they interact with an epithelium that is already structurally vulnerable, incompletely repaired, and prone to aberrant differentiation. In this setting, eosinophil-derived signals do more than intensify existing inflammation: they can weaken barrier integrity, redirect epithelial differentiation, alter repair trajectories, and sustain tissue remodeling [14,15] (Figure 1).

A central mechanism in this interaction is the induction of epithelial plasticity. Eosinophils promote an epithelial–mesenchymal transition (EMT)-like program in bronchial epithelial cells, with loss of E-cadherin, gain of vimentin, enhanced SMAD3 signaling, and increased transforming growth factor-β (TGF-β) 1 activity [16]. Moreover, eosinophils inhibit epithelial surface plasmin generation through TGF-β-dependent plasminogen activator inhibitor (PAI)-1 upregulation, and this change likely promotes fibrin retention and defective release rather than orderly repair of the damaged surface [17]. This remodeling propensity is biologically plausible in human diseases, where eosinophils are localized to compartments that matter most for epithelial dysfunction: airway eosinophilia correlates with poor epithelial integrity and basement membrane thickening, while intraepithelial eosinophils appear particularly relevant to AHR and type 2 inflammation [15,18].

Barrier damage is further exacerbated when eosinophils adhere to activated epithelium and degranulate. Classic studies have shown that eosinophil basic protein causes ciliostasis, cilia loss, epithelial breakdown, and epithelial cell detachment, thus establishing a direct link between eosinophil granule proteins and epithelial damage [18,19]. However, eosinophil granule proteins are not only cytotoxic, but they are also instructive remodeling signals. Eosinophil-derived cationic proteins can stimulate epithelial production of endothelin-1, TGF-α/β1, platelet-derived growth factor (PDGF), epidermal growth factor receptor (EGFR), matrix metalloproteinase (MMP)-9, fibronectin, and tenascin, indicating that eosinophils can convert damaged epithelial cells into a secondary source of profibrotic and proinflammatory mediators [8]. More recent work extends this concept by showing that eosinophil peroxidase (EPX) directly reprograms bronchial epithelial cells toward an early remodeling phenotype, increasing thymic stromal lymphopoietin (TSLP), TGF-β1, and disintegrin and metalloprotease 33 (ADAM33) expression and promoting EMT-like change through phosphatidylinositol 3-kinase/protein kinase B/mammalian target of rapamycin (PI3K/AKT/mTOR) signaling [20].

An additional layer of epithelial dysfunction is provided by eosinophil-derived cytokines. In mouse models of allergic asthma, eosinophils are the main source of IL-24, and genetic deletion or antibody neutralization of IL-24 reduces eosinophilic inflammation, AHR, collagen deposition, and mucus production [21]. Importantly, IL-24 directly compromises epithelial integrity—it lowers zonula occludens-1 (ZO-1), occludin, and tight junction protein 1 (TJP1) expression, increases epithelial permeability, and induces EMT-associated and profibrotic transcriptional programs in airway epithelial cells [21]. It also stimulates epithelial secretion of amphiregulin, granulocyte-macrophage colony-stimulating factor (GM-CSF), C-X-C motif chemokine 5 (CXCL5), IL-33, and TSLP, suggesting that eosinophils can reprogram the epithelium into a secondary amplifier of inflammation and remodeling rather than simply injure it. Although the strongest mechanistic evidence for this pathway currently comes from mouse and epithelial culture systems, the observation that circulating IL-24 is elevated in both children and adults with asthma supports its translational relevance [21].

Eosinophil extracellular traps (EETs) add another mechanism by which central eosinophil activation becomes persistent epithelial injury. These extracellular DNA-histone-granule protein scaffolds have been linked to epithelial desquamation, increased epithelial permeability, and increased epithelial release of IL-6 and IL-8 in severe eosinophilic asthma [22]. EETs also amplify epithelial alarm signaling, enhance type 2 innate immune activation, and activate pulmonary neuroendocrine cells to increase mucus secretion and neuro-immune signaling [22,23]. Furthermore, epithelial autoantigen-specific immunoglobulin G (IgG) may enhance EET formation in severe asthma, creating a feedback loop in which epithelial damage itself promotes further eosinophil activation [24]. Thus, eosinophil-induced barrier dysfunction is not limited to cell-contact toxicity; it may also be maintained extracellularly through DNA and protein networks that prolong the presence and biological activity of toxic eosinophil products in the airway lumen.

Charcot–Leyden crystals (CLCs) are closely linked to eosinophil cytolysis. They form when galectin-10, a cytoplasmic protein released from disrupted eosinophils, crystallizes in the extracellular space. Although CLCs were traditionally regarded as inert by-products, experimental studies have shown that they can amplify airway inflammation. Airway administration of crystalline galectin-10 activated innate and adaptive immune responses and enhanced type 2 inflammation and mucus production [25]. Antibodies that dissolved CLCs reduced these inflammatory effects [25]. Their persistence in eosinophil-rich airway mucus may therefore contribute to sustained epithelial activation, mucus hypersecretion, and further barrier injury. Sputum galectin-10 concentrations also correlate with sputum eosinophilia and may serve as a complementary marker of local eosinophilic inflammation [26].

Eosinophils also regulate epithelial differentiation toward a mucus-secretory phenotype. Co-culture studies show that eosinophils stimulate epithelial mucin 5AC (MUC5AC) production together with the release of profibrotic mediators such as TGF-β1, vascular endothelial growth factor (VEGF), and PDGF [27]. Analyses of human lung tissue support the relevance of this interaction: mucus-clogged asthmatic airways are enriched in proliferating basal cells and hypertrophic MUC5AC-positive goblet cells, and the plugs themselves are anchored to a remodeled, folded epithelial surface [28]. Once the epithelium has shifted into this MUC5AC-dominant state, the mucus becomes an instructive stimulus for eosinophils. Eosinophils exposed to IL-13-conditioned epithelial mucus undergo cluster of differentiation 11b (CD11b)- and glycan-dependent cytolytic degranulation, establishing a self-reinforcing loop between abnormal epithelial differentiation and eosinophil activation [28,29]. This mechanism may help explain why mucus plugs are linked to eosinophilia and airflow obstruction, why they can persist over time, and why chronic granulocytic plugs likely represent more than passive mucus retention [28,30,31].

At the same time, the interaction between eosinophils and epithelium is not exclusively destructive. Airway epithelial cells can recognize and engulf apoptotic eosinophils, suggesting that under favorable conditions, the epithelium may also participate in the resolution of eosinophilic inflammation [32]. In established asthma these restorative mechanisms appear to be outweighed by persistent eosinophil recruitment, degranulation, extracellular trap formation, cytokine release, and mucus-centered feedback loops. Overall, the most coherent synthesis is that eosinophils regulate epithelial dysfunction in asthma through several layered outputs—direct granule-protein toxicity, TGF-β-biased maladaptive repair, cytokine-mediated tight-junction destabilization, extracellular trap-driven amplification, and reinforcement of a MUC5AC-dominant secretory phenotype. The net result is a barrier-fragile, remodeling-prone epithelium that sustains inflammation, mucus retention, and progressive airflow impairment [14,15,21,28].

### 2.2. Chronic Eosinophilic Inflammation and Epithelial Remodeling in Asthma

Rather than representing an isolated inflammatory response, chronic eosinophilic inflammation in asthma is closely linked to a sustained epithelial–mesenchymal remodeling program. Within this framework, the airway epithelium functions as both the primary site of damage and an active coordinator of subsequent structural remodeling. Environmental triggers such as allergens, respiratory viruses, and pollutants disrupt epithelial integrity and induce the release of epithelial “alarmins,” particularly TSLP, IL-33, and IL-25, which amplify type 2 immunity and sustain eosinophilic inflammation [14,15,33,34,35,36,37].

A consistent message across the reviews is that airway remodeling is not simply a late consequence of severe disease. Epithelial injury, goblet-cell hyperplasia, reticular basement membrane thickening, extracellular matrix deposition, airway smooth-muscle expansion, and angiogenesis can be identified early in the disease course and may persist into adulthood [34,35,36,38]. This is important conceptually because it argues against viewing eosinophilia as a mere biomarker of severity. Instead, persistent eosinophilic inflammation appears to contribute directly to the conversion of repeated epithelial injury into abnormal repair, airway-wall thickening, and progressive airflow limitation [15,34,35,38].

Epithelium participates actively in this process by instructing both immune and mesenchymal cells. Asthmatic epithelial cells can drive fibroblasts toward increased expression of collagens, fibronectin, and TGF-β, thereby helping to establish a profibrotic microenvironment [34,39]. In parallel, epithelial-derived cytokines promote eosinophil recruitment and survival, while IL-33- and TSLP-dependent pathways strengthen mast-cell activation and type 2 cytokine production [33,35,37,40]. The consequence is a feed-forward loop in which epithelial dysfunction sustains eosinophilic inflammation, and eosinophilic inflammation in turn reinforces epithelial stress. Eosinophils themselves act as structural modifiers within this epithelial microenvironment. They localize to sites where remodeling is occurring, including the epithelial compartment, regions of poor epithelial integrity, and airways with thicker basement membranes and stronger mucus-repair phenotypes [14,15,33,41,42,43].

Eosinophils also push epithelial differentiation toward a mucus-secretory phenotype. Increased MUC5AC production, goblet-cell hyperplasia, and mucus retention are all closely linked to chronic eosinophilic inflammation and help explain why remodeled eosinophilic airways are prone to plugging and persistent airflow obstruction [14,15,27,33,35]. This mucus-centered remodeling pattern is not trivial: in chronic asthma, excess mucus reflects epithelial dysfunction and exacerbates the mechanical and inflammatory conditions that promote airway wall remodeling.

IL-5 biology may also provide an additional link between chronic eosinophilic inflammation and epithelial remodeling. The identification of functional IL-5 receptors on airway epithelial cells and fibroblasts suggests that IL-5 may contribute to structural airway changes not only indirectly, by supporting eosinophil survival, but also through direct effects on resident structural cells [15,44]. In keeping with this, IL-5-associated pathways have been linked to downregulation of epithelial tight-junction genes, while anti-IL-5 treatment has been associated with improved expression of epithelial transcripts involved in tight-junction pathways and ciliary organization [15,44,45]. Although the evidence that biologics reverse established remodeling remains incomplete, these observations support the broader idea that chronic eosinophilic inflammation is a modifiable driver of epithelial and airway-wall change rather than a passive correlate of disease activity [15,36,45].

The airway epithelium initiates a response to injury, but when eosinophils are attracted and retained, they help maintain epithelial dysfunction through alarmin amplification, TGF-β-biased plasticity, abnormal fibrinolysis, granule-protein signaling, mucus metaplasia, and ongoing interactions with fibroblasts, mast cells, and airway smooth muscle. The result is a self-reinforcing remodeled airway phenotype characterized by barrier fragility, abnormal repair, mucus hypersecretion, matrix accumulation, and in some patients a transition from variable obstruction to persistent airflow limitation [14,15,34,35,36,43]. For this reason, chronic eosinophilic inflammation should be regarded not only as an inflammatory endotype of asthma, but also as a structural disease-modifying process centered on the epithelium.

### 2.3. Eosinophils and Differentiation Dynamics in the Nasal Epithelium

The nasal epithelium represents a relevant upper-airway model for understanding how eosinophilic type 2 inflammation reshapes epithelial biology. Rather than functioning as a uniform barrier, the sinonasal mucosa consists of several interacting epithelial lineages, including basal, suprabasal, club, goblet, ciliated, and rarer secretory populations. Chronic type 2 inflammation disturbs this organization by altering both the distribution of these cell types and their normal differentiation trajectories [46,47]. This is particularly relevant in patients with asthma and coexisting chronic rhinosinusitis with nasal polyps (CRSwNP), in whom the upper and lower airways commonly share a pronounced eosinophilic type 2 inflammatory pattern [48,49,50,51]. In eosinophilic chronic rhinosinusitis these epithelial abnormalities coexist with intense eosinophilic infiltration, IL-5- and IL-13-rich inflammation, mucus hyperplasia, basement membrane thickening, and high recurrence rates, indicating that epithelial differentiation failure is a core feature of disease biology rather than a secondary epiphenomenon [48,49].

A recurring observation across histological and single-cell studies is that eosinophil-dominated disease disrupts normal epithelial repair. In CRSwNP, particularly in eosinophilic forms, the epithelial compartment tends to lose ciliated and club cell populations, while basal and goblet cell populations become more prominent. This shift suggests a transition away from effective mucociliary regeneration toward a more secretory and remodeling-prone epithelial state [46,47,52].

The clearest experimental evidence for this misdirected repair comes from studies of club-cell and basal-cell fate. In an IL-13-driven human nasal epithelial model, club cells were shown to be central to epithelial repair, yet exposure to IL-13 redirected secretoglobin 1A1 (SCGB1A1)-positive club/ciliated states toward SCGB1A1-positive, MUC5AC-positive mucus-secretory states [52]. Thus, one of the dominant downstream cytokines of eosinophilic inflammation appears to redirect epithelial regeneration away from ciliated recovery and toward goblet-cell metaplasia [48,49,52].

Inflammasome-related epithelial injury may further disturb basal-cell stability in CRSwNP. Activation of pyroptosis has been shown to impair basal-cell differentiation, reduce pathways associated with ciliated-cell development, and promote goblet-cell differentiation [53]. Although pyroptosis was present in both eosinophilic and noneosinophilic CRSwNP, the eosinophilic arm of the study identified IL-5 as a key upstream trigger, thereby placing a classic eosinophil-associated cytokine upstream of altered basal-cell fate [53].

A second major axis of differentiation change in eosinophilic polyp disease is EMT or, more broadly, epithelial dedifferentiation toward a remodeling phenotype. In eosinophilic CRSwNP, epithelial E-cadherin is reduced, vimentin is increased, and the degree of vimentin-positive epithelial reprogramming correlates with both eosinophil infiltration and disease severity [54]. These observations are strengthened by mechanistic work showing that IL-4 drives EMT-like change in primary human nasal epithelial cells through signal transducer and activator of transcription 6/interferon regulatory factor 4 (STAT6/IRF4) signaling [55]. In that model, IL-4 also polarized macrophages toward an M2-like state, increased TGF-β1 production, and amplified EMT in co-cultured epithelial cells. Epithelial dedifferentiation in eosinophilic disease is therefore sustained through a broader type 2 tissue circuit involving epithelial cells, macrophages, and TGF-β-rich remodeling signals [54,55].

Single-cell studies add a critical spatial and systems-level perspective to these processes. In CRSwNP, apical epithelial populations decrease progressively from healthy tissue toward polyp tissue, whereas glandular epithelial populations expand, especially in tissue adjacent to polyps [56]. These epithelial changes occur alongside restructuring of the fibroblast network toward states capable of sustaining chemotaxis, extracellular matrix deposition, and chronic polyp growth [47,56].

The eosinophils themselves are also more complex than the older literature suggested. Single-cell RNA sequencing of eosinophils isolated from nasal polyps demonstrated that tissue eosinophils differ markedly from blood eosinophils and separate into multiple activated subclusters within polyp tissue [57]. These eosinophil states were enriched for cytokine-mediated signaling, nuclear factor kappa-light-chain-enhancer of activated b cells (NF-κB)-related programs, anti-apoptotic pathways, growth-factor activity, and molecules consistent with adhesion and intercellular communication [57]. Clinical studies involving patients with severe eosinophilic asthma and coexisting nasal polyposis support this united-airway perspective. Mepolizumab improved both sinonasal and asthma outcomes in this phenotype [50]. Similarly, in the ANDHI nasal polyposis substudy, benralizumab improved sinonasal symptoms together with asthma exacerbation rates, lung function, and asthma control [51]. These findings support the clinical relevance of a shared IL-5/eosinophil axis in the upper and lower airways, although these studies assessed clinical outcomes rather than direct restoration of epithelial differentiation.

Overall, the most coherent interpretation of the available evidence is that eosinophils are not simply downstream effector cells in nasal polyp disease. They participate in the regulation of epithelial differentiation dynamics by shaping the cytokine milieu, amplifying epithelial-immune and epithelial-stromal crosstalk, and helping maintain a chronically altered repair niche. Within that niche, basal and club cells are redirected away from durable mucociliary restoration, goblet-cell programs expand, EMT-like reprogramming intensifies, and fibroblast-engaging epithelial states become more prominent [48,49,50,51,52,53,54,55,56,57,58]. Thus, in patients with asthma and eosinophilic CRSwNP, abnormalities of nasal epithelial differentiation may be regarded as part of a broader eosinophil-dominated airway disease, while recognizing that the nasal epithelium is not a complete surrogate for the bronchial epithelium.

## 3. Biomarkers of Epithelial Injury: A Window into Airway Epithelial Barrier Status

The previous sections showed that epithelial dysfunction in asthma, particularly in eosinophilic and type 2-high disease, is not simply a consequence of inflammation. It is also an important part of disease development and persistence. The airway epithelium is involved in several processes, including maintenance of barrier integrity, innate immune responses, inflammatory cell recruitment, and tissue repair. When these functions become disrupted, changes occur in airway structure, immune responses, epithelial differentiation, and remodeling pathways [6,13]. Epithelial injury is also not limited to structural damage alone. Epithelial cells release signals that maintain type 2 inflammation and promote eosinophil recruitment and persistence. These signals can also affect repair processes and contribute to structural airway changes rather than normal restoration of barrier function [3,59].

**Figure 2:**
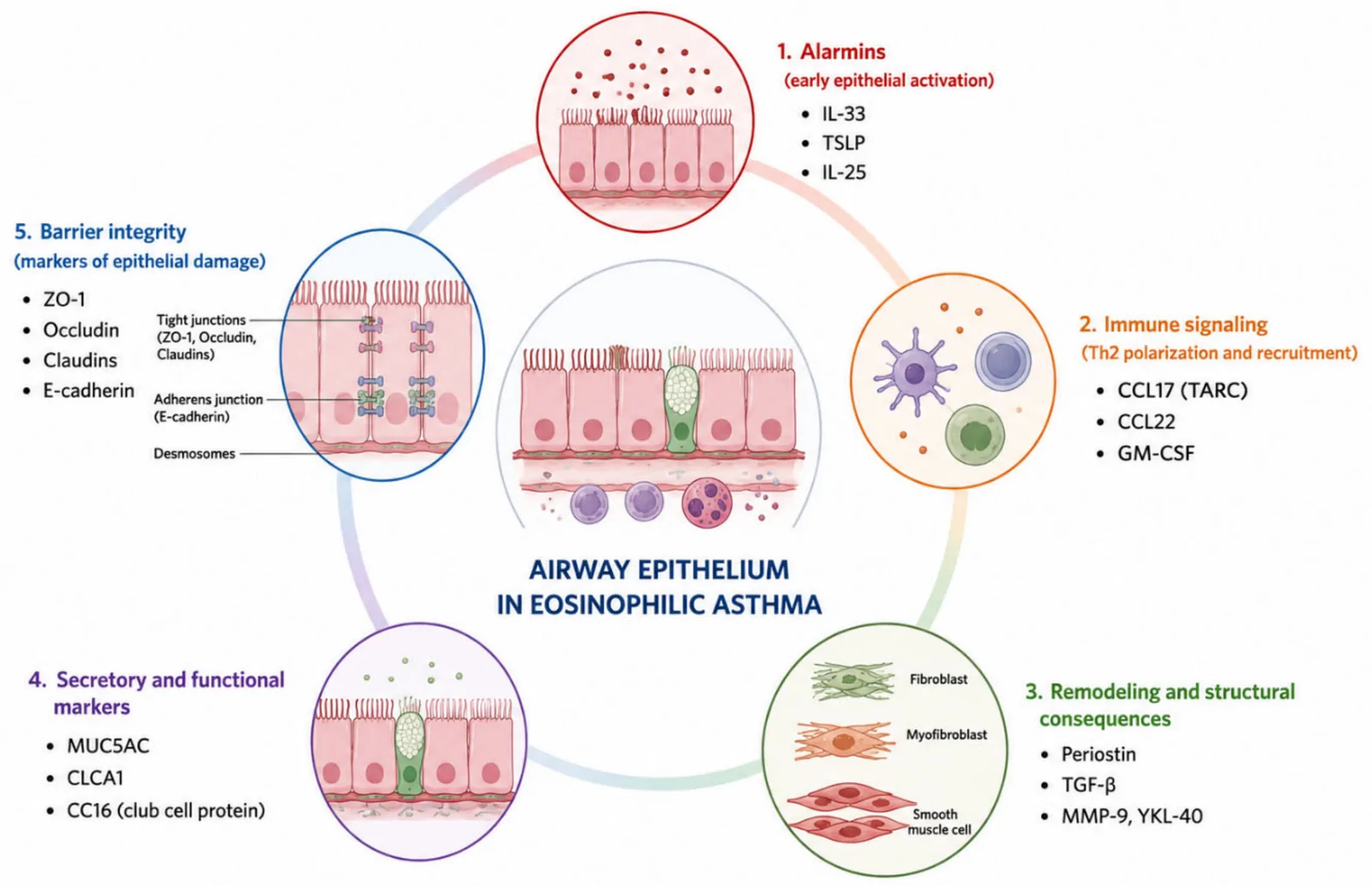
Key epithelial biomarkers associated with airway epithelial dysfunction in eosinophilic asthma. The figure summarizes major biomarker groups reflecting airway epithelial activation, immune signaling, remodeling, epithelial differentiation, and barrier integrity. Alarmins (IL-33, TSLP, and IL-25) represent upstream epithelial activation, whereas CCL17, CCL22, and GM-CSF are involved in type 2 immune signaling. Periostin, TGF-β, MMP-9, and YKL-40 are associated with airway remodeling. MUC5AC, CLCA1, and CC16 reflect epithelial differentiation and secretory function. Structural barrier markers, including ZO-1, occludin, claudins, and E-cadherin, indicate epithelial junction integrity and barrier function. CCL, C-C chemokine ligand; CC16, Clara cell protein; CLCA, Calcium-Activated Chloride Channel Regulator; GM-CSF, granulocyte macrophage colony-stimulating factor; IL, interleukin; MMP, matrix metalloproteinase; MUC5AC, mucin 5AC; TGF-β, transforming growth factor beta; TSLP, thymic stromal lymphopoietin; YKL-40, Chitinase-3-like protein 1; ZO-1, zonula occludens-1.

Biomarkers of epithelial injury should be considered in a broader context. They do not only reflect tissue damage and may also represent different biological changes occurring in the airways. Some biomarkers are linked to early epithelial activation and immune signaling, whereas others are associated with later changes such as mucus hypersecretion, extracellular matrix remodeling, and barrier disruption [60,61] (Figure 2). This becomes particularly relevant in eosinophilic asthma. Interactions between epithelial cells and eosinophils occur in both directions. Epithelial-derived mediators contribute to eosinophilic inflammation, while eosinophils themselves promote epithelial injury and remodeling through cytotoxic granule proteins, cytokine release, and extracellular trap formation [14,15].

### 3.1. Epithelial-Derived Alarmins: Upstream Drivers of Eosinophilic Inflammation

Among epithelial biomarkers, IL-33, TSLP, and IL-25 are involved in early stages of the inflammatory response and connect epithelial injury with type 2 inflammation. These cytokines are released by epithelial cells after exposure to allergens, viral infections, pollutants, and other environmental triggers. They activate dendritic cells, type 2 innate lymphoid cells, and T helper 2 (Th2) lymphocytes [59,62]. As a result, production of IL-5 and IL-13 increases, promoting eosinophil differentiation, recruitment, and survival.

Increased expression of IL-33 has been reported in bronchial biopsies from patients with asthma and has been associated with AHR and type 2 inflammatory activity [40,63]. TSLP expression is also increased in the airway epithelium and has been linked to disease severity and persistent type 2 inflammation [37]. Both IL-33 and TSLP appear to play an important role in eosinophilic asthma and may also be useful as biomarkers. IL-25 is involved in similar pathways, although available clinical data remain more limited and much of the evidence comes from mechanistic and translational studies rather than patient cohorts [59].

IL-33 and TSLP have also been studied as markers of epithelial activation. Increased levels have been found in bronchial tissue, sputum, nasal epithelium, and other airway samples and have been associated with disease severity, AHR, and type 2 inflammatory patterns [37,63]. TSLP has also gained attention as a therapeutic target, as blockade of this pathway reduces exacerbation frequency across different asthma phenotypes [62]. At the same time, circulating levels of these alarmins can vary considerably because their release often occurs locally within the airways. This may limit their use as isolated systemic biomarkers.

### 3.2. Epithelial Activation and Immune Signaling: Coordination of Type 2 Inflammation

A second group of biomarkers is related to the role of the airway epithelium in immune cell recruitment and regulation of inflammatory responses. Chemokines such as chemokine (C-C) ligand (CCL) 17 and CCL22 are involved in recruitment of C-C motif chemokine receptor 4 (CCR4)-positive Th2 lymphocytes and promote production of IL-4, IL-5, and IL-13 [64]. However, the cellular sources of these chemokines differ. Bronchial epithelial cells are a recognized source of CCL17. In contrast, epithelial CCL22 expression has been demonstrated mainly under specific inflammatory conditions, such as rhinovirus infection, and epithelial cells are not considered a predominant source of CCL22 in the airways [64]. In the airways, CCL22 is produced predominantly by myeloid immune cells, including monocytes, dendritic cells, and macrophages [65,66]. Recruitment of Th2 lymphocytes and the resulting cytokine production contribute to persistent type 2 inflammation and ongoing eosinophilic responses.

Epithelial cells also appear to contribute to differences between asthma endotypes. Transcriptomic studies have linked epithelial-derived mediators with AHR and variability in inflammatory patterns [8,42]. Their role, therefore, may extend beyond simple inflammatory markers.

Increased levels of CCL17 and CCL22 have been reported in type 2-high asthma and are associated with eosinophilic inflammation in both airway and blood samples. However, CCL17 may partly reflect cytokine-driven epithelial activation, whereas CCL22 should be interpreted as a marker of broader airway immune-cell activation rather than as an epithelial-specific biomarker [64,66]. Their specificity, however, remains limited because increased levels can also occur in other allergic and inflammatory diseases. C-X-C motif chemokine ligand 8 (CXCL8, IL-8) and IL-6 are more often associated with neutrophilic or mixed inflammatory phenotypes and are therefore less useful as biomarkers of eosinophilic asthma [3]. GM-CSF contributes to eosinophil survival but is produced by different cell types and lacks specificity for eosinophilic inflammation. Plasminogen activator inhibitor-2 (*SERPINB2*, PAI-2) has been identified in epithelial gene signatures associated with type 2 inflammation and may reflect IL-13-related epithelial activation, although its association with eosinophilic inflammation appears indirect [67]. CCL28 participates in mucosal immune responses, but evidence supporting its use as a biomarker in eosinophilic asthma remains limited [68].

### 3.3. Stress, Repair, and Remodeling Markers: Structural Consequences of Persistent Eosinophilic Inflammation

Markers of epithelial stress, repair, and remodeling reflect structural airway changes that occur during chronic inflammation. Periostin is one of the most studied biomarkers in type 2-high asthma. It is induced by IL-13 and has been linked to eosinophilic inflammation, subepithelial fibrosis, and airway remodeling [69]. Higher periostin concentrations have also been observed in more severe disease and have been explored as predictors of response to biologic treatment targeting IL-13 and IL-5 pathways.

Eosinophils are involved in airway remodeling through several mechanisms. One of them is the release of transforming growth factor-β (TGF-β), which contributes to extracellular matrix deposition and epithelial–mesenchymal transition-like changes [34,35]. MMP-9 reflects matrix degradation and remodeling activity, while chitinase-3-like protein 1 (*CHI3L1*, YKL-40) has been linked to chronic airway inflammation and structural changes in asthma [70]. Increased levels of MMP-9 and YKL-40 are not limited to eosinophilic asthma and can also be found in other inflammatory phenotypes.

Amphiregulin has been linked to epithelial repair and type 2 inflammatory responses. It may also reflect tissue remodeling. Its expression is not limited to eosinophilic inflammation, which reduces its usefulness as an isolated biomarker [71]. Overall, markers within this group are best interpreted as indicators of chronic epithelial reprogramming and structural disease rather than specific markers of eosinophil-driven inflammation.

### 3.4. Secretory Proteins: Functional Readouts of Epithelial Differentiation

Secretory proteins are associated with epithelial differentiation and functional changes under type 2 inflammatory conditions. IL-13-driven changes in the airway epithelium promote goblet cell metaplasia and increase expression of MUC5AC, an important mucin involved in mucus hypersecretion in eosinophilic asthma [28,72]. Increased MUC5AC expression has been reported in severe asthma and has been associated with altered epithelial differentiation, mucus plugging, and airflow obstruction [28,30].

Calcium-activated chloride channel regulator 1 (CLCA1) is often co-expressed with MUC5AC and is associated with mucus-producing epithelial phenotypes, suggesting a role in type 2-related epithelial changes [72]. Increased expression of these markers has also been linked to mucus plugging, airflow limitation, and persistent disease activity.

Mucin 5B (MUC5B) differs from MUC5AC and is more closely related to normal airway mucus production, with less specific association with type 2 inflammation [72]. Club cell secretory protein (CCSP16, CC16) is more closely linked to epithelial integrity and repair. Lower levels have been reported in epithelial injury and impaired lung function [73]. Unlike MUC5AC or CLCA1, CC16 is not directly related to eosinophilic inflammation.

### 3.5. Structural Barrier Integrity Markers: Indicators of Epithelial Damage

Structural barrier integrity markers include claudins, occludin, ZO-1, and E-cadherin, which are components of tight and adherens junctions and contribute to maintenance of the epithelial barrier [60]. Airway epithelial cells from patients with asthma show reduced expression and altered localization of these junctional proteins together with impaired barrier function [60,61,74].

Cytotoxic granule proteins and extracellular traps released by eosinophils have been linked to disruption of epithelial junctions and increased epithelial permeability [22,23]. Similar changes are also reported in other inflammatory conditions and are not restricted to eosinophilic inflammation. These markers therefore reflect epithelial barrier injury more than eosinophilic activity itself.

### 3.6. Clinical Relevance of Epithelial Injury Biomarkers in Induced Sputum and Peripheral Blood

Induced sputum allows airway inflammation and epithelial injury to be assessed in the same sample. Eosinophilic inflammation can be determined by differential cell counts, while shed columnar epithelial cells may indicate epithelial damage. Qin et al. identified a columnar epithelial cell-high phenotype that was more common in severe asthma and associated with non-neutrophilic airway inflammation [75]. Thus, this phenotype is not specific to eosinophilic asthma. However, samples with both eosinophilia and high epithelial cell counts had increased CLCA1 expression and periostin concentrations, indicating concomitant type 2 inflammation and epithelial activation [75]. Combined measurement of sputum eosinophils, epithelial cells, and epithelial products may distinguish eosinophilic inflammation accompanied by epithelial injury from eosinophilia alone. As induced sputum does not require bronchoscopy, it may also be suitable for repeated assessment. Further validation and standardized methods for epithelial cell quantification are required before its routine clinical use.

Among the biomarkers discussed above, periostin, YKL-40, and CC16 have the most direct evidence supporting their assessment in peripheral blood. In a recent pediatric study, serum periostin was associated with blood eosinophilia, FeNO, and exercise-induced bronchoconstriction, whereas YKL-40 was related to impaired lung function and methacholine responsiveness [76]. In adults with severe asthma, lower serum CC16 concentrations were associated with sputum eosinophilia, a lower FEV1/FVC ratio, and a more rapid decline in FEV1 over five years [77]. IL-25, IL-33, and TSLP can also be detected in serum, and changes in their concentrations have been observed during mepolizumab treatment [78]. CCL17 and CCL22 have likewise been quantified in plasma, although evidence supporting their clinical use remains exploratory and is based on small patient cohorts [79]. At present, none of these circulating mediators is validated as a specific marker of epithelial injury or restoration; therefore, they should be regarded as complementary rather than stand-alone biomarkers [76,77,78,79].

Different groups of biomarkers can be identified in epithelial injury associated with eosinophilic asthma. Some are linked with type 2 inflammatory pathways, while others are related to structural changes and tissue repair. Not all of them directly reflect eosinophilic activity. Several biomarkers represent broader changes occurring in the airway epithelium, including epithelial activation, Th2 responses, and airway remodeling. These biomarkers may provide different types of information. Markers related to epithelial signaling can reflect more active disease processes that may still be reversible, whereas remodeling markers are more often associated with persistent tissue changes. Understanding which epithelial abnormalities improve and which remain over time may help in assessment of biological remission and treatment selection in asthma. The biological samples and analytical methods used to assess these biomarkers are summarized in Table 1.

## 4. From Inflammation Control to Epithelial Restoration and Clinical Remission

### 4.1. Targeting Eosinophilic Inflammation: Implications Beyond Symptom Control

The introduction of biologic therapies targeting type 2 inflammation has changed the way severe asthma is treated, with greater emphasis now placed on targeting the underlying mechanisms of disease. In eosinophilic asthma, monoclonal antibodies directed against the IL-5 pathway reduce eosinophil activity and their persistence within tissues, targeting one of the key mechanisms involved in disease pathogenesis [9,80]. Clinical trials and real-world studies demonstrate that these therapies reduce exacerbation frequency, improve asthma control, and decrease oral corticosteroid dependence, supporting the role of eosinophil-targeted therapy in the management of severe eosinophilic asthma [81,82,83].

Eosinophils affect not only inflammatory processes but also epithelial injury and structural airway changes. Eosinophilic inflammation has been closely linked to airway remodeling processes, including epithelial damage, extracellular matrix deposition, and airway smooth muscle alterations [84]. Through the release of cytotoxic mediators and inflammatory signals, eosinophils may drive epithelial damage and altered repair mechanisms [85]. Molecular studies have shown that biologic therapies reduce eosinophil-related gene expression, while some epithelial inflammatory pathways may remain active [86], indicating that reduction of eosinophilia alone may not fully restore epithelial function. Early clinical studies suggest that eosinophil-targeted therapy may also influence epithelial signaling pathways. Anti-IL-5 treatment has been associated with reduced circulating levels of epithelial-derived alarmins, such as IL-25 and TSLP, which suggests that suppression of eosinophils may also reduce epithelial inflammatory activity [87].

**Table 1:** Summary of biomarkers of airway epithelial activation and damage in asthma.

Biomarker(s)	Biological Relevance	Sample Types	Analytical Methods	Refs.
IL-33	Epithelial alarmin released during cellular stress or injury; upstream type 2 activation	Bronchial biopsies and primary bronchial epithelial cells; serum	IHC; RT-qPCR; ELISA or multiplex immunoassay	[59,63,78]
TSLP	Epithelial alarmin and upstream regulator of type 2 inflammation	Bronchial biopsies or epithelial samples; serum	IHC or IF; RT-qPCR; ELISA	[37,59,78,87]
IL-25	Epithelial alarmin associated with type 2 inflammation and eosinophilic activity	Bronchial epithelial samples; serum	IHC or IF; RT-qPCR; ELISA	[59,78,87]
CCL17	CCR4-dependent Th2-cell recruitment; may reflect cytokine- or virus-induced epithelial activation	Nasal and bronchial secretions; primary bronchial epithelial cell cultures; plasma	Multiplex immunoassay; RT-qPCR; ELISA	[64,79]
CCL22	CCR4-dependent Th2-cell recruitment; predominantly reflects myeloid-cell activation	Nasal and bronchial secretions; primary bronchial epithelial cell cultures	Multiplex immunoassay; RT-qPCR; ELISA	[64,65,66]
CXCL8, IL-6, GM-CSF	Epithelial and immune-cell activation; more closely related to neutrophilic or mixed inflammation	Induced sputum; BAL fluid; serum/plasma; bronchial epithelial cell cultures	ELISA or multiplex immunoassay; RT-qPCR or RNA-seq	[3,59,67]
SERPINB2 (PAI-2)	IL-13-responsive epithelial gene signature; indirect marker of type 2 epithelial activation	Bronchial epithelial brushings; induced sputum cells	RT-qPCR; microarray analysis or RNA-seq	[67]
CCL28	Mucosal chemotaxis and epithelial immune signaling; limited evidence as an asthma biomarker	Primary human airway epithelial cells; A549 epithelial cells; culture supernatants	RT-qPCR; protein immunoassay	[68]
Periostin	IL-13-related epithelial activation, eosinophilic inflammation, and extracellular matrix remodeling	Serum; induced sputum supernatant	ELISA or automated immunoassay	[69,75,76]
TGF-β1, MMP-9	Abnormal repair, matrix deposition or degradation, and airway remodeling; not epithelial-specific	Induced sputum; BAL fluid; bronchial tissue	ELISA or other immunoassays; IHC; gelatin zymography for MMP-9	[34,35]
YKL-40	Inflammation and airway remodeling; not specific to eosinophilic or epithelial activity	Serum	ELISA	[70,76]
Amphiregulin	Epithelial repair and tissue remodeling; produced by epithelial and immune cells	Plasma; saliva; PBMCs; bronchial biopsies; primary bronchial epithelial cells and culture supernatants	ELISA; RT-qPCR; IHC	[71]
MUC5AC, MUC5B	Goblet-cell differentiation and mucus production; MUC5AC is more closely related to type 2 inflammation	Sputum and mucus plugs; BAL fluid; bronchial tissue or epithelial brushings	RT-qPCR or RNA-seq; immunostaining or immunoblotting; proteomic analysis	[28,30,72]
CLCA1	IL-13-responsive mucus-secretory epithelial phenotype	Induced sputum cells; bronchial epithelial brushings	RT-qPCR; microarray analysis or RNA-seq	[72,75]
CC16 (SCGB1A1)	Club-cell secretory function and epithelial integrity; lower concentrations may indicate epithelial injury	Serum; BAL fluid; induced sputum; airway epithelial tissue or cells	ELISA or automated immunoassay; RT-qPCR; IHC	[73,77]
Claudins, occludin, ZO-1, E-cadherin	Tight- and adherens-junction integrity; reduced expression or altered localization indicates barrier disruption	Bronchial biopsies or brushings; nasal epithelial brushings; primary airway epithelial cultures	IHC, IF, or confocal microscopy; RT-qPCR; Western or in-cell Western blotting; TEER and permeability assays	[60,61,74]
Galectin-10/CLCs	Eosinophil cytolysis and local eosinophilic activity; crystalline galectin-10 can amplify type 2 inflammation and secondary epithelial injury	Induced sputum; patient-derived airway CLC preparations in mechanistic studies	Cytospin microscopy and semiquantitative Western blotting for sputum galectin-10; crystal imaging and structural analysis	[25,26]
Sputum columnar epithelial cells	Epithelial shedding and injury; interpretation is strengthened by concurrent inflammatory-cell counts	Induced sputum	Cytospin microscopy and differential cell counts; flow cytometry; epithelial gene-expression analysis	[75]

BAL, bronchoalveolar lavage; CCL, C-C motif chemokine ligand; CC16, club cell secretory protein 16; CLCs, Charcot–Leyden crystals; CLCA1, calcium-activated chloride channel regulator 1; CXCL8, C-X-C motif chemokine ligand 8; ELISA, enzyme-linked immunosorbent assay; GM-CSF, granulocyte-macrophage colony-stimulating factor; IF, immunofluorescence; IHC, immunohisto-chemistry; IL, interleukin; MMP-9, matrix metalloproteinase-9; MUC5AC/MUC5B, mucin 5AC/5B; PAI-2, plasminogen activator inhibitor-2; PBMCs, peripheral blood mononuclear cells; RT-qPCR, reverse-transcription quantitative polymerase chain reaction; RNA-seq, RNA sequencing; SCGB1A1, secretoglobin family 1A member 1; SERPINB2, serpin family B member 2; TEER, transepithelial electrical resistance; TGF-β1, transforming growth factor-β1; TSLP, thymic stromal lymphopoietin; YKL-40, chitinase-3-like protein 1; ZO-1, zonula occludens-1. Sample types and methods are based on the cited primary studies and reviews. For grouped biomarkers, not every analyte was evaluated in every listed sample or by every listed method. Several mediators are also produced by immune or mesenchymal cells; therefore, detection in a given sample does not establish epithelial specificity. Most listed markers remain investigational and are not validated as stand-alone clinical measures of epithelial injury.

### 4.2. Clinical Remission and Its Biological Limitations

Clinical remission has become an important treatment goal in the era of biologic therapy for asthma. It is commonly described as sustained symptom control, no exacerbations, no need for systemic corticosteroids, and stable lung function [88]. Real-world and clinical trial data shows that some patients treated with biologics can achieve remission, although the reported rates differ and are usually around 30–40%, depending on the criteria used [82,89]. Similar results have been reported in long-term studies of anti-IL-5 treatment, where only some patients reached complete remission. Around one-quarter to one-third met strict remission criteria despite improvements in eosinophil counts, lung function, and asthma control [78].

Long-term observational studies also suggest that remission may persist over time. Patients treated with benralizumab have shown sustained remission during longer treatment periods, although responses vary across different populations [83,90]. Many patients achieve improvements in some aspects of disease control, such as reduced exacerbation frequency, without meeting full remission criteria [89].

Even when clinical remission is achieved, some disease mechanisms may remain active. Epithelial dysfunction, airway remodeling, and ongoing inflammatory signaling can persist despite stable clinical symptoms. Structural and epithelial changes are not always fully reversed after suppression of eosinophilic inflammation, particularly in patients with long-standing disease [84,91]. Clinical remission should not be considered the same as complete biological recovery. Changes in type 2 mediators after treatment also appear uneven. Levels of IL-5, IL-13, and TSLP decrease with treatment, whereas epithelial-derived cytokines such as IL-25 may remain unchanged, suggesting that epithelial activation can persist despite effective eosinophil suppression [78].

### 4.3. Impact of Biologics on Airway Remodeling and Epithelial Structure

Airway remodeling plays an important role in the relationship between persistent airway inflammation and long-term disease progression in asthma. Structural abnormalities such as epithelial damage, subepithelial fibrosis, goblet cell hyperplasia, and airway smooth muscle hypertrophy are commonly seen in severe asthma and contribute to ongoing airflow limitation [84].

Recent studies suggest that biologic therapies targeting eosinophilic inflammation may affect not only inflammatory activity but also structural changes in the airways. Eosinophils contribute to several processes involved in airway remodeling and are an important source of profibrotic mediators, including TGF-β, which is involved in fibroblast activation, extracellular matrix production, and airway smooth muscle growth. In addition, IL-5 signaling may directly affect structural airway cells, suggesting that its role in airway remodeling is not limited to regulating eosinophil survival [92]. These findings have also been supported by studies examining airway tissue. Mepolizumab treatment has previously been associated with reductions in reticular basement membrane thickness and airway smooth muscle mass [91]. More recently, the MESILICO study provided further evidence that anti-IL-5 therapy may influence airway structure [92]. After 12 months of mepolizumab treatment in patients with severe eosinophilic asthma, reductions were observed in sub-basement membrane thickness, airway smooth muscle area, tissue eosinophilia, and epithelial damage, accompanied by improvements in lung function and asthma control. The reduction in airway smooth muscle thickness was also associated with a decrease in tissue eosinophils, supporting the relationship between eosinophilic inflammation and structural airway changes.

Results for other biologic therapies have been more variable. For example, benralizumab has been associated with possible reductions in airway smooth muscle burden, although supporting evidence is still limited [11]. Even when inflammatory markers improve, structural changes in the airways may persist, suggesting that inflammation control alone may not be sufficient for complete restoration of airway structure [92].

At the molecular level, biologic therapies alter gene and protein expression profiles associated with inflammation and remodeling. Systematic analyses show consistent reductions in eosinophil-related pathways but more heterogeneous effects on epithelial and structural networks [85].

Mucus pathology provides another example of persistent epithelial dysfunction in asthma. Mucus plugging, associated with airflow obstruction and type 2 inflammation, reflects epithelial reprogramming and goblet cell metaplasia. Although biologic therapies may reduce mucus burden, changes in epithelial pathways involved in mucus production may persist, indicating that epithelial recovery may remain incomplete [86]. At the cellular level, anti-IL-5 therapy has been associated with reduced eosinophil activity and lower levels of proteins that contribute to epithelial injury and airway remodeling [93].

### 4.4. Biomarkers of Treatment Response and Epithelial Recovery

Biomarkers play an important role in selecting biologic therapies and evaluating treatment response. Commonly used markers such as blood eosinophil count and fractional exhaled nitric oxide (FeNO) are widely used to identify type 2 inflammation and predict response to biologic treatment [80]. However, these markers do not fully reflect the complexity of asthma biology. Recent studies have shown that novel serum biomarkers, including soluble IL-5 receptor α, pentraxin-3, thioredoxin-1, and extracellular matrix degradation products, may reflect different aspects of disease biology, such as inflammation, oxidative stress, and airway remodeling. Available data indicates that combining different biomarkers may improve the assessment of disease activity [94].

Commonly used biomarkers may not fully reflect changes within the airways, particularly epithelial dysfunction and airway remodeling [95]. This limitation is particularly relevant for remission evaluation, as these markers mainly reflect inflammatory activity rather than epithelial repair or restoration of airway structure.

Recent transcriptomic studies have shown that biologic therapies can change inflammatory gene expression patterns, but changes in epithelial-related pathways appear to be less consistent [86]. These observations point to the importance of combining inflammatory, epithelial, and structural markers when assessing disease activity and treatment response.

### 4.5. From Inflammation Suppression to Epithelial Restoration: Current Evidence and Future Directions

Current evidence suggests that reducing eosinophilic inflammation alone may not be enough for full epithelial recovery. Although biologic therapies effectively reduce inflammation and improve clinical outcomes, their impact on epithelial repair and restoration of airway structure may be incomplete. The concept of disease modification has therefore gained increasing attention. Biologics may exert early anti-inflammatory effects and later structural effects, with potential long-term influence on airway remodeling depending on disease stage and treatment timing [84]. However, complete reversal of established structural changes remains uncertain.

Molecular studies suggest that epithelial abnormalities and non-type 2 inflammatory pathways may persist despite eosinophil-targeted therapy [86]. The persistence of these changes may partly explain why some patients continue to have incomplete responses despite improvements in inflammation. Clinical remission and biological remission are therefore not necessarily the same. Current therapies can effectively control inflammation, but recovery of epithelial function and airway structure may require earlier treatment, more precise biomarkers, and therapeutic approaches that target epithelial repair in addition to inflammation.

## 5. Conclusions

Eosinophilic asthma is a chronic disease in which interactions between eosinophils and the airway epithelium contribute to the development and persistence of asthma. These interactions can lead to epithelial barrier dysfunction, altered epithelial differentiation, and abnormal repair processes. Inflammation affects the airway epithelium, although the epithelium itself also contributes to disease processes. Environmental triggers, type 2 inflammation, and airway remodeling are closely linked. Eosinophils contribute through release of cytotoxic mediators, cytokines, extracellular traps, and profibrotic factors, leading to epithelial injury, mucus overproduction, and structural airway changes.

Biomarkers related to epithelial injury reflect different aspects of these processes. Some are associated with epithelial activation, including IL-33 and TSLP, whereas others are linked with mucus production, airway remodeling, or disruption of epithelial junctions. Not all biomarkers directly reflect eosinophilic inflammation. Some represent broader epithelial changes and may provide information beyond commonly used inflammatory markers.

Biologic therapies targeting eosinophilic inflammation have improved clinical outcomes, and remission is now achieved in some patients. Still, reduction of eosinophilia does not always result in complete recovery of epithelial structure and function. Epithelial abnormalities and structural airway changes can remain present even in clinically stable patients. Clinical remission and biological remission do not necessarily represent the same condition.

Restoring epithelial barrier function and normal epithelial differentiation are emerging goals in asthma treatment. Control of inflammation alone may not be sufficient in all patients, and strategies directed at epithelial repair may also be needed. Broader biomarker approaches may also improve assessment of disease activity and remission. Better understanding of these biological processes may help move asthma management beyond symptom control and toward more targeted treatment strategies.

## Data Availability

No new data were created or analyzed in the preparation of this review. Data sharing is not applicable to this article.

## Abbreviations

The following abbreviations are used in this manuscript:

ADAM33	A disintegrin and metalloprotease 33
AHR	Airway hyperresponsiveness
AKT	Protein kinase B
CCL	C-C chemokine ligand
CC16 (CCSP16)	Club cell secretory protein 16
CCR4	C-C motif chemokine receptor 4
CD11b	Cluster of differentiation 11b
CHI3L1	Chitinase-3-like protein 1
CLCs	Charcot–Leyden crystals
CLCA1	Calcium-activated chloride channel regulator 1
CRSwNP	Chronic rhinosinusitis with nasal polyps
CXCL5	C-X-C motif chemokine ligand 5
CXCL8	C-X-C motif chemokine ligand 8
EETs	Eosinophil extracellular traps
EGFR	Epidermal growth factor receptor
EMT	Epithelial–mesenchymal transition
EPX	Eosinophil peroxidase
FeNO	Fractional exhaled nitric oxide
GM-CSF	Granulocyte-macrophage colony-stimulating factor
IgG	Immunoglobulin G
IL	Interleukin
IRF4	Interferon regulatory factor 4
MMP-9	Matrix metalloproteinase-9
mTOR	Mammalian target of rapamycin
MUC5AC	Mucin 5AC
MUC5B	Mucin 5B
NF-κB	Nuclear factor kappa-light-chain-enhancer of activated B cells
PAI-1	Plasminogen activator inhibitor-1
PDGF	Platelet-derived growth factor
PI3K	Phosphatidylinositol 3-kinase
SCGB1A1	Secretoglobin family 1A member 1
SERPINB2	Serpin family B member 2
SMAD3	SMAD family member 3
STAT6	Signal transducer and activator of transcription 6
TGF-α	Transforming growth factor alpha
TGF-β	Transforming growth factor beta
Th2	T helper 2
TJP1	Tight junction protein 1
TSLP	Thymic stromal lymphopoietin
VEGF	Vascular endothelial growth factor
YKL-40	Chitinase-3-like protein 1
ZO-1	Zonula occludens-1
